# The Rectal Stump During and After Subtotal Colectomy for Ulcerative Colitis: A Narrative Review of Surgical Strategies, Medical Management Options, and Cancer Surveillance Recommendations

**DOI:** 10.3390/jcm15031114

**Published:** 2026-01-30

**Authors:** Orestis Argyriou, Itai Ghersin, George Stravodimos, Guy Worley, Phil Tozer, Ailsa Hart, Kapil Sahnan

**Affiliations:** 1 Department of Colorectal Surgery, St Mark’s the National Bowel Hospital and Academic Institute, London NW10 7NS, UK; 2 Department of Surgery and Cancer, Imperial College London, London W12 0NN, UK; 3 Department of Gastroenterology, St Mark’s the National Bowel Hospital and Academic Institute, London NW10 7NS, UK; 4 Department of Colorectal Surgery, Chelsea and Westminster Hospital NHS Foundation Trust, London SW10 9NH, UK; 5 Department of Colorectal Surgery, Royal London Hospital, Barts Health NHS Trust, London E1 1FR, UK; 6 Department of Metabolism, Digestion and Reproduction, Imperial College London, London W12 0NN, UK

**Keywords:** colectomy, colitis, ulcerative, proctitis, rectal stump

## Abstract

**Background/Objectives**: There are multiple decision nodes, during and after subtotal colectomy for ulcerative colitis (UC), regarding the management of the rectal stump. Intra-operatively, the surgeon must decide on the closure technique and positioning of the retained stump, while post-operatively, clinicians often face the challenge of managing diversion proctitis, as well as determining an appropriate endoscopic surveillance strategy, given the increased risk of cancer. This narrative review aims to summarise the evidence relating to these key decision points in rectal stump management. **Methods**: A narrative review of the literature was performed. Relevant studies were identified through a search of Ovid Medline and Embase. Inclusion criteria were adult population and diagnosis of UC. Cohort studies, review articles, and guidelines were eligible for inclusion. The references were grouped according to the subject of interest and reported accordingly. **Results**: Intra-peritoneal closure has been shown to have higher pelvic sepsis rates (5–25%), whereas subcutaneous placement results in higher rates of wound infections (up to 15%). A mucous fistula has been shown to have the lowest overall complication rate. Microscopic findings compatible with diversion proctitis develop in most patients, with incidence ranging from 71.4% to 100%. However, only a minority of these patients (30–40%) develop symptoms. Suggested treatments for diversion proctitis include topical mesalamine, corticosteroids, or short-chain fatty acids. The overall risk of rectal stump neoplasia in patients with UC after subtotal colectomy is as low as 0.7%, with prior colorectal neoplasia being a major risk factor. No universal standardised guidance exists regarding endoscopic surveillance in this patient population. **Conclusions**: This narrative review has appraised the latest evidence on three crucial stages of rectal stump management in UC. There is still uncertainty about the optimal surgical management of the stump, with different complication profiles. Medical management of diversion proctitis remains a major unmet need, and there are no randomised trials addressing this issue. There are no universally accepted guidelines on endoscopic surveillance of the rectal stump.

## 1. Introduction

Ulcerative colitis (UC) is a chronic, relapsing–remitting inflammatory bowel disease (IBD) that affects the colon. Approximately 5–10% of UC patients will have surgery within 5 years of diagnosis, most frequently in the form of a colectomy and end ileostomy [1]. The main indications for a subtotal colectomy include acute severe colitis (ASUC), medically refractory disease, and the presence of dysplastic or malignant changes [2]. For acute severe colitis or complications, a subtotal colectomy is performed in the emergency setting, whereas in cases of failure of medical management or dysplasia/cancer, this is performed electively or in an expedited fashion.

At subtotal colectomy, the colon is resected, while the rectum remains in situ, as a stump, and an end ileostomy is constructed.

The formation and subsequent management of the rectal stump encompass a range of decision nodes, both intra-operatively and post-operatively.

Intra-operatively, three main options are described: closure of the stump intraperitoneally, closure of the stump at the level of the rectus sheath, or formal exteriorisation as a mucous fistula. The decision regarding operative stump management involves balancing subsequent patient symptoms and the safety profile from infective complications (intra-abdominal sepsis/collection, in cases of leak of an intra-peritoneal stump or wound infection/collections in the cases of closure at the level of the sheath) [3]. The importance of reporting on rectal stump blow-out, as part of the Key Performances Indicators (KPIs) for surgical service provision, has also been highlighted through Pan-European Delphi Consensus by the European Crohn’s and Colitis Organisation (ECCO) [4].

Post-operatively, microscopic evidence of proctitis (due to residual disease or diversion) is present in the majority of UC patients with a retained rectal stump. Some of these patients develop symptoms of proctitis, such as tenesmus, mucous discharge, or haematochezia, that require addressing. The medical management of diversion proctitis remains controversial [5].

Furthermore, the increased risk of colorectal cancer in UC remains relevant, as these patients are still at risk of developing dysplastic changes in the retained rectal stump. This necessitates endoscopic surveillance of the stump, with the recommended frequency still a matter of debate [6].

**Figure 1:**
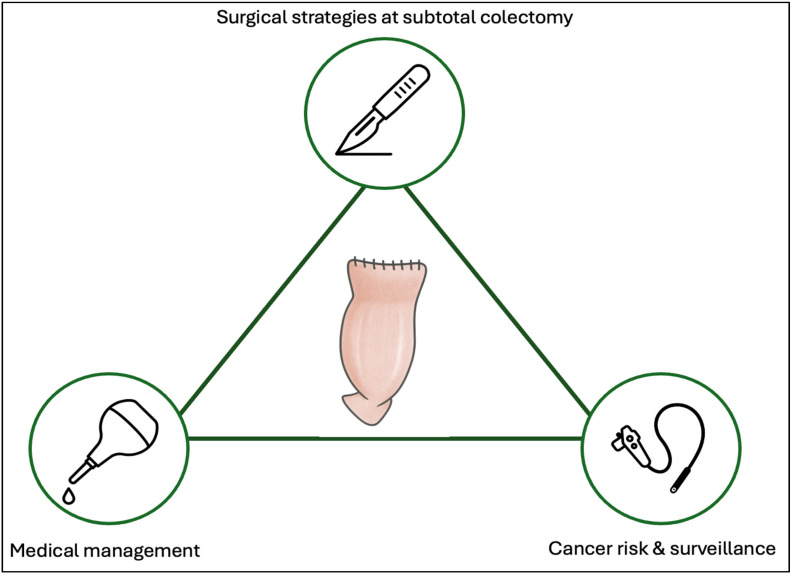
Main decision nodes in rectal stump management.

The aim of this narrative review is to summarise and discuss all available evidence on these three aspects in the management of the rectal stump in UC (Figure 1), while also highlighting recommendations from the most recent national and international guidelines.

## 2. Materials and Methods

A comprehensive search strategy was created, and searches of Ovid Medline and Embase were conducted on the 19 June 2025, with the use of the following search terms: ulcerative colitis OR subtotal colectomy OR colectomy OR stump OR proctitis OR cancer OR surveillance.

Given the aim of widely exploring all aspects of rectal stump management, the following inclusion criteria were applied:

- Studies in English.
- Studies including adult populations only.
- Studies including patients diagnosed with UC (and reporting separately on them, if part of a larger cohort/group).
- Studies of any form: cohorts, review articles, guidelines, and conference abstracts.

Articles were screened and reviewed by 3 independent reviewers (OA, IG, and GS), and conflicts were resolved by a 4th reviewer (KS). For synthesis, the references were grouped according to the subject of interest. No statistical analyses were performed.

Generative artificial intelligence (GenAI) was used to generate Figure 1 with ChatGPT (GPT-5; OpenAI, San Francisco, CA, USA). The authors take responsibility over the content generated.

## 3. Results

**1.**  **Intra-operative management of the stump (during subtotal colectomy)**

**Overview of Strategies and Considerations**

The three main management strategies for the rectal stump at subtotal colectomy are:

- Division of the rectum above or at the level of the peritoneal reflection, without exteriorising or securing at the sheath.
- Placement of a closed stump in the subcutaneous tissue, after securing at the rectus sheath.
- Exteriorisation of the stump, at the level of the skin, as a mucous fistula.

Relative variability in practice has been captured previously: in a 2022 survey study of 20 colorectal centres, by the Italian society of colorectal surgery (SICCR), intraperitoneal closure was the preferable method (15 centres), followed by subcutaneous placement (3 centres) and mucous fistula formation (2 centres) [7].

Another emerging surgical option, described by Mege et al. (2020), proposes the formation of an ileosigmoidostomy, where the sigmoid mucous fistula is exteriorised at the same stoma site as the end ileostomy [8]. This seems to be equally safe and effective, when compared with intraperitoneal closure (102 vs. 212 patients, respectively), particularly in view of future two- or three-stage reconstruction with ileal pouch-anal anastomosis (IPAA). Notably, there was no incidence of stump leak in the comparator group, appreciating though that all cases were retrospectively identified as performed IPAAs, and a stump leak may have prevented restorative surgery.

The formation of a mucous fistula seems to have the safest profile, since the retained colonic stump is exteriorised and opened. Comparison of the remaining two options aims to mainly mitigate the risk and burden of complications; leak or blow-out of an intra-peritoneal stump may require a re-operation, frequently in the form of a laparotomy, whereas the same leak, at the level or above the sheath, may be managed with a wound washout and result in a controlled fistula. Disease burden and activity, as well as the potential of subsequent surgery (ileal-pouch anal anastomosis—IPAA, ileorectal anastomosis—IRA, or completion proctectomy), must also be considered.

The options of a closed rectal stump in the subcutaneous tissue and that of a mucous fistula dictate a longer rectal (or distal sigmoid) stump, therefore leaving a diseased segment in situ. When the stump is closed intraperitoneally, this may be left short or long, to facilitate future surgery.

As a result, it is of the outmost importance to examine surgical strategies and outcomes in relation to the indication for colectomy; both in relation to disease activity or burden, and the physiological state of the patient (electively for refractory disease versus in an emergency fashion for complications, such as toxic megacolon).

Another key consideration, when reviewing the literature, is a relative lack of clear definitions and descriptions of complications. Studies may generally report on pelvic sepsis or collection rates, without specifying whether this was a result of leak or blow-out of an intraperitoneal stump. Similarly, wound infection rates need to be described in relation to the subcutaneous stump.

**Latest Evidence**

The latest systematic review addressing surgical strategies was performed by Bedrikovetski et al. in 2019 [9]. The authors identified 11 papers, spanning from 1991 to 2013, either describing the results and outcomes of one strategy, or comparing two. Intraperitoneal closure (examined in seven studies) was found to have a pelvic sepsis rate of 5.3% and a wound infection rate of 7.9%. Subcutaneous placement (examined in five studies) was found to have a pelvic sepsis rate of 2% and a wound infection rate of 14.5%. The presence and rates of pelvic sepsis in subcutaneous placement and wound infection in intraperitoneal closure highlight the previously made point about clear definitions and correlations to the surgical options.

Nevertheless, in this systematic review, at least five of the eleven included studies appear to have some limitations and heterogenous characteristics, regarding the emergency of the procedure, the included population, or the rates of each technique. In more detail, in the study by Bohm et al., comparing mucous fistula formation with intraperitoneal closure, in 31 subtotal colectomies for UC performed acutely or electively, complication rates are not reported separately for the acute/emergency indications [10]. In the largest study, by Gu et al., of 204 colectomies for ASUC, comparing intraperitoneal versus subcutaneous closure, cases of colitis associated with massive haemorrhage, colonic perforation, or toxic megacolon were excluded [11]. Most importantly, in the largest intraperitoneal closure cohort, by Brady et al. (159 patients), on subtotal colectomies for both emergency (85.5%) and elective indications, there is no separate reporting of complications (overall pelvic sepsis due to stump dehiscence or pelvic collection 6.9%) [12]. Trickett et al., in a study comparing intra-peritoneal closure with subcutaneous placement in 37 emergency subtotal colectomies, do not distinguish complication rates of each option in relation to diagnosis (34 UC, 2 Crohn’s colitis, and 1 infective colitis) [13]. Lastly, the study by Pal et al., reports a 6.6% rate of stump blow-out, in a cohort of 60 emergency UC subtotal colectomies, where the stump was either closed intra-peritoneally or exteriorised as a mucous fistula, without specifying the respective rates [14]. This heterogeneity, in addition to the power of each study (with the number of patients in each ranging from 14 to 204), limits the evidence basis.

Useful conclusions may also be drawn from another recent systematic review and meta-analysis, by Lawday et al. (2020) [15], that looked at rectal stump management for any IBD indication. In this review, there was overlap of nine papers that were included in the Bedrikovetski review [9], with the remaining five either being published earlier than 1991, or including mixed or Crohn’s populations. The meta-analysis reported an overall rectal stump leak rate (both intra-peritoneally and subcutaneously) of 4.9% (95% CI 3.7–6.6), being lower in mucous fistula (2.4%, 95% CI 0.8–7.3) and highest in the subcutaneous group (12.6%, 95% CI 8.3–18.6). Pelvic abscess/sepsis was found to have an incidence rate of 5.7% (95% CI 4.4–7.3), being higher with intraperitoneal closure (11.1%, 95% CI 5.8–20.3).

Studies undertaken after the publication of these systematic reviews demonstrate similar rates, but with the already appreciated limitations. Buschs et al. reported a 3.3% stump leak rate (with 1.3% requiring re-operation), in a study of 151 UC subtotal colectomies, comprising both elective (51) and emergency cases (100) [16]. In a large study of 307 subtotal colectomies for any UC indication, with almost equal numbers for intraperitoneal and subcutaneous closure, Lissel et al. reported a 7% rate of stump leak in intraperitoneal closure and a 33% rate of wound problems in relation to the stump, in the subcutaneous placement group [17]. Similarly, in a study of 180 UC subtotal colectomies with intra-peritoneal closure, for any elective or emergency indication, Schineis et al. reported a 6.1% stump leak rate [18]. In their own cohort analysis, Lawday et al., reported an 8.3% stump leak rate, in 51 intraperitoneal stumps, out of a 61-patient cohort, including subtotal colectomies for any indication (emergency and elective) and any diagnosis (UC and CD) [15]. In a smaller study of 38 subtotal colectomies for ASUC, Renaud et al. reported only one case of stump leak (2.6%) [19]. Lastly, as a general reference, Ritter et al. investigated risk factors for stump leak for any discontinuity resection (benign and malignant) in a large cohort of 713 patients, including 53 for IBD diagnoses (UC in 37), reporting an overall stump leak rate of 11.78%, with a 24.3% rate in UC cases [20].

Preliminary results of more studies have been presented as conference abstracts and report leak rates in accordance with the existing literature. Huber et al. reviewed 411 patients, with UC or unspecified IBD, that underwent subtotal colectomy prior to IPAA creation reporting 15 cases of rectal stump leak (3.65%), with 7 requiring a reoperation [21]. Kumar et al. compared stump-related morbidity in a 164-patient cohort, for both IBD and non-IBD indications, reporting a statistically significant higher stump leak rate in IBD patients (0.05% vs. 0.01%, *p* = 0.032) [22]. In a study of 117 patients with medically refractory UC undergoing colectomy, Ozcimen et al. reported a 9% stump leak rate, with 6% of these having been placed subcutaneously [23].

The results of the Management in Acute Severe Colitis (MASC) audit, by the European Society of Coloproctology (ESCP), are eagerly awaited. The design of this audit will allow for better comparison of the stump closure techniques, focusing on emergency indications and stratifying by disease severity, physiological status and other intra-operative parameters. Preliminary results of 258 surgical patients, presented at the 2023 ECCO conference, reported a 65% rate of intra-peritoneal closure, 20% of no stump (panproctocolectomy or ileo-anal pouch formation), 9% of mucous fistula, and 3% of subcutaneous placement [24]. Full results, with subgroup analysis on stump leak rates and wound infections, will certainly provide a strong evidence basis, capturing European practices and variations.

**Other Intra-Operative Considerations (During Subtotal Colectomy)**

Apart from the management of the rectal stump, surgical decision-making at the time of subtotal colectomy includes additional decision nodes, which are appreciated below.

*Surgical Approach*

Minimally invasive surgery (MIS) approaches, either laparoscopically or robotically, have been shown to result in shorter lengths of stay and lower risk of infectious complications [16,25].

Although MIS is strongly supported and encouraged by various guidelines [26], it is still acknowledged that the availability of MIS depends on local expertise, whereas the patient’s condition (physiological state and haemodynamic instability) and presence of complications (perforation and ischaemia) may dictate an open approach.

*Level of Vascular Transection*

Another important intraoperative consideration at subtotal colectomy is the level of vascular transection. The surgeon must decide between high ligation of the inferior mesenteric artery (IMA) or preservation of the IMA pedicle and superior rectal vessels (and subsequently allowing for a longer rectal stump). The latter approach keeps the pelvic anatomy undisrupted, facilitates subsequent proctectomy or restorative surgery (by maintaining ‘virgin’ rectal dissection planes), and reduces the risk of pelvic nerve injury [27].

A survey of Italian centres by Mineccia et al. [7] demonstrated a lack of consensus regarding the level of vascular transection. Four centres (20%) reported transecting the IMA at its origin, nine centres (45%) transected the mesenteric trunk distally, at the level of the sigmoid artery, and seven centres (35%) performed distal ligation, close to the bowel wall.

In the presence or suspicion of dysplasia or cancer, naturally, dissection and vascular transection should be performed in an oncologic fashion, with ligation close to the origin.

*Drainage of the Rectal Stump*

The use of a rectal drain is described in the literature and is particularly common in the setting of a closed intraperitoneal rectal stump, where it aims to reduce the risk of stump blowout and subsequent septic complications. This is based on the hypothesis that rectal staple line failure in some cases may be a consequence of increased intrarectal pressure, due to retained rectal secretions. Consequently, decompression of the rectal remnant using transanal catheter drainage can reduce or prevent this complication.

In a 114-patient cohort of colectomies for any IBD colitis, of which 36% had a rectal drain placed, Karch et al. reported an overall 2.6% pelvic sepsis rate (that occurred only in patients without a drain) [28]. In a historic study by Fleshner et al., reporting on 14 colectomies post cyclosporin therapy failure, a large-bore rectal drain was placed in 10/14 cases, with one case of rectal stump dehiscence (from the group that had a drain) [29].

The aforementioned survey of practices in Italy examined this topic as well, with nine centres (45%) performing a transrectal drainage to prevent the risk of rectal stump dehiscence blowout, six centres (30%) not treating the stump in any way, and five (25%) managing with topical medications [7]. Transanal drainage is also discussed in the Practice Parameters for the Surgical Treatment of Ulcerative Colitis by The American Society of Colon and Rectal Surgeons (ASCRS); it is noted that if an intraperitoneal closed stump is preferred, then transanal drainage of the distal stump may further decrease the risk of pelvic sepsis [30].

Overall, this still remains at the surgeon’s discretion, with intra-operative assessment of the stump (degree of inflammation/friability) being key for this decision.

**Overview of National and International Guidelines on Intra-Operative Rectal Stump-Related Considerations**

The 2018 Association of Coloproctology of Great Britain and Ireland (ACPGBI) guidelines, on surgery for IBD, provide a weak grade of recommendation (D), based on a low level of evidence (IV), highlighting that the management of the stump remains controversial and at the surgeon’s discretion, mentioning that a stump leak at the subcutaneous level is a less morbid complication [31]. The 2017 ECCO guidelines on diagnosis and management of UC do not make a specific recommendation, but discuss thoroughly the management of the rectal remnant at subtotal colectomy, highlighting the safety profile of subcutaneous closure and mucous fistula, advising rectal drainage for cases of intraperitoneal closure, to prevent blow-out [32]. The 2021 American Society of Colon and Rectal Surgeons (ASCRS) guidelines do not make a strong recommendation; the options of subcutaneous closure, mucous fistula and rectal drainage are recommended to mitigate the risk of stump dehiscence [33]. Lastly, the 2025 British Society of Gastroenterology (BSG) guidelines recommend a subtotal colectomy with an end ileostomy and a long rectal stump, for medically refractory disease, without specifying the closure technique [34].

**Management of Post-Operative Rectal Stump Blow-Out**

Lavryk et al. have proposed a management algorithm, for rectal stump blow-out after colectomy for IBD [35]. The algorithm starts by physical and biochemical findings, and incorporates all potential surgical options, decision nodes, and management options, including interventional and operative management.

**The Impact of Pre-Operative Medications on Stump-Related Complications**

*Corticosteroids*

Pre-operative corticosteroid exposure is a well-recognised risk factor for impaired wound healing and infective complications, and is therefore biologically plausible as a contributor to rectal stump leak and subsequent pelvic sepsis following subtotal colectomy. While there is no standardised guidance that links specific steroid thresholds to a mandated stump strategy, existing surgical principles and guideline recommendations (as above) emphasise selecting an approach with the most favourable safety profile in higher-risk settings; in practice, where concern for stump-related sepsis is increased (including in the context of significant steroid exposure), this may strengthen the consideration of strategies such as exteriorisation as a mucous fistula rather than leaving an intraperitoneal closed stump, where a leak may manifest as intra-abdominal/pelvic sepsis.

*Biologics*

In contrast, available evidence does not consistently demonstrate an increased risk of postoperative complications in patients exposed to biologic therapy prior to colectomy for ulcerative colitis. For example, Ward et al. reported no association between recent anti-TNF exposure and postoperative complications after subtotal colectomy (in 6225 colectomies, of which 753 were receiving anti-TNF) [36], and Kim et al. similarly found no significant difference in overall 30-day postoperative complications between vedolizumab-, anti-TNF-, and biologic-naïve groups (in a matched 153 patient cohort) [37]. Taken together, these data do not suggest that biologic exposure alone should drive stump strategy selection, although interpretation remains limited by heterogeneity in study design, exposure timing/definitions, and outcome reporting.

Importantly, these considerations are particularly relevant in the ASUC setting, as most patients proceeding to colectomy will have received systemic corticosteroids immediately prior to surgery, reinforcing the need to account for pre-operative medication exposure when counselling and when selecting the safest stump management technique.

**Considerations for Future Surgery, in Relation to the Rectal Stump (After Subtotal Colectomy)**

*Preparation of the Stump, Prior to Restorative Surgery*

For patients considering restorative surgery, eligibility must be thoroughly assessed, taking into account rectal compliance and severity of proctitis (mostly for IRA) and sphincter function (for IPAA) [38]. Notably, pre-IPAA-formation rectal stump inflammation has been correlated with worse IPAA outcomes [39]. Nevertheless, no formal guidance or consensus recommendations exist on how best to prepare the rectal stump for restorative surgery. Generally, it is considered desirable to reduce mucosal inflammation and address diversion proctitis before reconstruction, using measures that follow the general principles of diversion proctitis management, as described below. However, there is no agreed preoperative regimen, either regarding agents or duration.

*Caesarean Section, in the Presence of a Rectal Stump*

At present, there is very limited evidence to guide the choice of delivery mode (vaginal delivery or caesarean section—CS) in women who become pregnant after subtotal colectomy for UC, with a retained rectal stump. Current pregnancy and delivery guidelines in IBD do not identify this situation as an indication for CS over vaginal delivery—in contrast to women with an existing or planned IPAA, for whom CS is often recommended to protect pelvic floor and anal sphincter function [40,41].

When a CS is required for obstetric reasons, it is important to obtain a detailed operative history, including whether the rectal stump was closed intraperitoneally or brought out subcutaneously (as a mucous fistula will usually be evident on examination). If intraperitoneal closure was performed, the risk profile is not substantially different from that of any patient with previous abdominal surgery and potential adhesions. In contrast, a subcutaneously placed stump carries a risk of inadvertent injury at the time of CS, and these cases should be assessed and planned by a multidisciplinary team including colorectal surgeons and obstetricians to minimise that risk.

**Further Work**

To allow for clear comparison of the surgical options, according to indications and disease severity, further research needs to be undertaken. This should be planned prospectively, with clear definitions and detailed data collection for stump leak/dehiscence and wound infection rates, matched to the relevant technique. Data collection and analysis should incorporate clearly the indication for colectomy, as well as the disease severity.

**2.**  **Diversion proctitis in the retained stump**

Diversion proctitis is thought to develop due to the lack of nutrients from luminal bacteria to the rectal mucosa. The most important nutrients are thought to be short-chain fatty acids, such as butyric acid. The lack of these oxidative substrates leads to inflammation and cellular damage [42]. Microscopic findings compatible with diversion proctitis develop almost universally in the rectal stump after surgical faecal diversion, in both IBD and non-IBD patients, with incidence ranging from 71.4% to 100% [43,44,45].

However, only a minority of these patients (30–40%) develop symptoms [5], with symptoms including tenesmus, rectal bleeding, or mucous discharge, and can lead to a decreased quality of life [10]. Endoscopic features of diversion proctitis include friable mucosa (even with minimal air insufflation), oedema, erythema, nodularity, exudates, and ulcers [46].

In current clinical practice, differentiation between diversion proctitis and residual active IBD of the rectal stump remains a significant challenge. Symptoms alone are usually insufficient, as they overlap substantially across these entities. However, the presence of concomitant extra-intestinal manifestations may point towards active IBD rather than diversion proctitis. The endoscopic appearance is also often indistinguishable. However, features such as deep ulcerations favour a diagnosis of active IBD. Histologically, although many features are non-specific [47,48], findings such as diffuse lymphoid hyperplasia [49], lymphocytic phlebitis [50], and granulomatous vasculitis favour diversion proctitis over active IBD, whereas transmural inflammation, well-formed granulomas, and fissuring ulcers support Crohn’s disease of the rectal stump.

In non-IBD patients, the best treatment for diversion proctitis is surgical restoration of faecal continuity. In IBD, however, this option is not advisable for a large proportion of patients. Thus, topical anti-inflammatory agents are the cornerstone of treatment in these cases. Suggested therapies for diversion proctitis include topical mesalamine, corticosteroids, or short-chain fatty acids. Unfortunately, their efficacy has not been established by randomised trials. Evidence supporting the use of these therapies is limited to case series and small retrospective studies [5]. Adequately powered randomised controlled trials are sorely required to establish the efficacy of the various suggested medical treatment for diversion proctitis.

**3.**  **Dysplasia/Cancer risk and Surveillance strategies in the retained stump**

Colorectal cancer is one of the most dreaded complications of long-standing UC [51]. Dysplastic lesions might develop in the retained rectal stump of UC patients as well. Rectal stump malignancies pose a unique diagnostic challenge. In patients with a rectal stump, rectal bleeding and discharge secondary to diversion proctitis are relatively common. These symptoms can mimic or mask an underlying malignancy.

The overall risk for rectal stump neoplasia in patients with UC after subtotal colectomy appears to be quite low. A 2016 meta-analysis estimated a pooled rectal carcinoma prevalence of 2.1% [51]. This contrasts with a 2023 systematic review, reporting a rectal carcinoma prevalence of 0.7% in this patient cohort [52].

When translated into incidence rates, available cohort data suggest an absolute cancer risk in the order of approximately 0.1–0.3 cases per 100 patient-years, although estimates vary widely due to differences in study design, follow-up duration, and patient selection. This is equivalent to one rectal stump cancer per 333–1000 patient-years.

The higher reported prevalence in the older meta-analysis might be explained by selection bias, as it included mainly single-centre studies.

Prior colorectal neoplasia is consistently identified as the major risk factor for neoplasia in patients with IBD and a retained rectum [53]. The above-mentioned 2023 systematic review [52] reported an adjusted HR of 5.1 (95% CI 3.1–8.2) for developing rectal stump carcinomas in patients with pre-operative dysplasia, and 7.2 (2.4–21.1) for patients with pre-operative colorectal cancer. One study identified PSC and disease duration until colectomy as risk factors for rectal stump cancer [54].

No universal standardised guidance exists regarding endoscopic surveillance in this patient population [6]. The 2021 consensus guideline by the Global Interventional Inflammatory Bowel Disease Group on endoscopic evaluation of surgically altered bowel in inflammatory bowel disease suggests that patients at risk of dysplasia should undergo yearly surveillance endoscopy of the diverted rectum [55]. They define patients at risk of dysplasia as those with a preoperative diagnosis of colitis-associated neoplasia, a long history (>8 years) of UC, concurrent primary sclerosing cholangitis (PSC), rectal strictures, or a family history of colorectal cancer in a first-degree relative diagnosed at age 50 years or younger. This guideline does not make rectal stump surveillance recommendations for patients without the above-mentioned risk factors. It makes specific recommendations regarding biopsies during surveillance endoscopy of the rectal stump, suggesting four random biopsies every 10 cm should be taken, using a modified Seattle protocol. Furthermore, any visible lesion should be biopsied or completely removed for histology.

On the other hand, the ECCO 2023 Guideline on Inflammatory Bowel Disease and Malignancies suggests that patients at risk of dysplasia, defined as disease duration ≥ 8 years, prior colorectal cancer, or concomitant PSC, should undergo endoscopic surveillance of the rectal stump every two years [56]. For patients without these risk factors, surveillance endoscopies at least every 5 years were recommended. The technical aspect of biopsy-taking during surveillance endoscopy of the rectal stump was not addressed in this guideline.

The ACPGBI 2018 guideline on surgery for inflammatory bowel disease mentions previous history of colorectal cancer and duration of disease prior to colectomy as risk factors for rectal stump malignancy [31]. It suggests patients with a rectal stump should have regular surveillance of the rectal stump, and that the frequency of surveillance should be based on the above-mentioned risk factors. It does not specify the surveillance intervals for patients at risk of dysplasia nor patients without these risk factors. It also does not address the technical aspect of biopsy-taking during surveillance endoscopy of the rectal stump.

All the above-mentioned guidelines base their recommendations on low-quality evidence and expert opinions. There is a need for higher-quality studies on this subject, addressing the risk factors for rectal stump malignancy and the outcomes of different rectal stump surveillance strategies. These may provide the evidence base required for the creation of uniform universal guidelines on the surveillance of this unique patient population.

## 4. Discussion

Management of the rectal stump in patients undergoing subtotal colectomy for ulcerative colitis has critical decision points with major implications for outcomes.

The wide range of surgical strategies, extending from intraperitoneal closure to subcutaneous placement and mucous fistula formation, emphasises the tailored approach to treatment based on clinical status and severity of the disease. It would seem that evidence supports the finding that intraperitoneal closure is associated with a relatively increased risk of pelvic sepsis, whereas subcutaneous placement results in a higher rate of wound infection. Mucous fistula formation has the most favourable complication profile. However, it is often less acceptable to patients because it requires management of a second ostomy. In addition, it may not always be clinically feasible. Standardisation of definitions and improved reporting of outcomes would help in providing clearer insights on the rates of complications according to each surgical approach.

Rectal stump management is further compounded in the postoperative period by complications like diversion proctitis and the possibility of dysplastic changes in the retained rectal stump. As noted above, there are no randomised controlled trials to guide the medical treatment of diversion proctitis, and this remains a major unmet need.

Societal guideline recommendations vary regarding optimal rectal stump surveillance intervals, as noted above. Based on contemporary incidence estimates, the risk of rectal stump malignancy is relatively low (0.1–0.3 rectal stump cancers per 100 patient-years). Therefore, hundreds of surveillance endoscopies are likely to be required to detect a single malignancy in an unselected population, with the number needed to scope likely substantially higher in patients without recognised risk factors. In this context, the balance between benefit and harm becomes uncertain. While endoscopic surveillance is generally safe, it is not without risks, costs, patient discomfort, and psychological burden [57,58,59], particularly in diverted rectal stumps affected by chronic inflammation and friability. Notably, the extension of surveillance recommendations to low-risk patients appears to be driven largely by extrapolation from colitis-associated cancer paradigms and by medico-legal considerations [60], rather than by direct evidence demonstrating improved clinical outcomes in this subgroup.

These observations emphasise the need for a risk-stratified approach to rectal stump surveillance, prioritising patients with clearly defined high-risk features such as preoperative dysplasia/cancer or concomitant PSC. Until prospective data demonstrate a tangible benefit from routine surveillance in low-risk patients, clinicians should engage in shared decision-making that transparently communicates the low absolute cancer risk, the uncertain benefit of surveillance, and the cumulative procedural burden.

We believe the most critical unmet need in rectal stump management is the absence of a standardised, evidence-based management pathway. This knowledge gap spans multiple domains, including uncertainty regarding optimal intra-operative stump management, the limited evidence base for medical treatment of diversion proctitis, and the lack of a universally accepted, risk-stratified surveillance strategy for rectal stump neoplasia.

To address these deficiencies, we strongly advocate for the establishment of a prospective, international rectal stump registry. Such a registry would provide high-quality data on short-, intermediate-, and long-term outcomes and enable meaningful risk stratification.

We envision a registry capturing outcomes at three predefined time points:

(1) An immediate postoperative node, focusing on surgical complications.
(2) An intermediate node at 1–3 years, capturing diversion proctitis and patient-reported outcomes (PROs).
(3) A long-term node at ≥5 years, primarily focused on oncologic surveillance and malignancy outcomes.

Importantly, PROs should be considered core endpoints in future rectal stump studies. There is growing recognition in inflammatory bowel disease that traditional clinician-reported outcomes and endoscopic findings incompletely capture disease burden [61,62,63]. Symptoms commonly associated with a diverted rectum, such as urgency, tenesmus, rectal pain, and mucous discharge, can have a profound impact on daily functioning and quality of life [64,65], and frequently matter more to patients than outcomes such as endoscopic appearance or surgical complications. Incorporating validated PRO measures into rectal stump research is therefore essential to ensure that future management strategies are aligned with patient priorities and deliver patient-centred care.

## 5. Conclusions

This narrative review has appraised the latest evidence on three crucial stages of rectal stump management in UC. There is still uncertainty about the optimal surgical management of the stump, with different complication profiles. Medical management of diversion proctitis remains a major unmet need, and there are no randomised trials addressing this issue. There are no universally accepted guidelines on endoscopic surveillance of the rectal stump. There is a pressing need for high-quality prospective trials to better elucidate these areas of controversy.

## Data Availability

No new data were created.

## References

[1] Zhao M., Gönczi L., Lakatos P.L., Burisch J. (2021). The Burden of Inflammatory Bowel Disease in Europe in 2020. J. Crohns Colitis.

[2] Worley G.H.T., Vaughan-Shaw P., Sahnan K. (2023). Surgical Management of Ulcerative Colitis. Br. J. Surg..

[3] Landerholm K., Wood C., Bloemendaal A., Buchs N., George B., Guy R. (2018). The Rectal Remnant after Total Colectomy for Colitis–Intra-Operative, Post-Operative and Longer-Term Considerations. Scand. J. Gastroenterol..

[4] Morar P.S., Hollingshead J., Bemelman W., Sevdalis N., Pinkney T., Wilson G., Dunlop M., Justin Davies R., Guy R., Fearnhead N. (2017). Establishing Key Performance Indicators [KPIs] and Their Importance for the Surgical Management of Inflammatory Bowel Disease—Results from a Pan-European, Delphi Consensus Study. J. Crohns Colitis.

[5] Dal Buono A., Carvello M., Sachar D.B., Spinelli A., Danese S., Roda G. (2021). Diversion Proctocolitis and the Problem of the Forgotten Rectum in Inflammatory Bowel Diseases: A Systematic Review. United Eur. Gastroenterol. J..

[6] Derikx L.A.A.P., De Jong M.E., Hoentjen F. (2018). Short Article: Recommendations on Rectal Surveillance for Colorectal Cancer after Subtotal Colectomy in Patients with Inflammatory Bowel Disease. Eur. J. Gastroenterol. Hepatol..

[7] Mineccia M., Perotti S., Pellino G., Sampietro G.M., Celentano V., Rocca R., Daperno M., Ferrero A. (2022). Emergency Colectomy for Acute Severe Ulcerative Colitis: A Nationwide Survey on Technical Strategies of the Italian Society of Colorectal Surgery (SICCR). Updates Surg..

[8] Mege D., Frontali A., Pellino G., Adegbola S., Maggiori L., Warusavitarne J., Panis Y. (2020). Laparoscopic Subtotal Colectomy with Double-End Ileosigmoidostomy in Right Iliac Fossa Facilitates Second-Stage Surgery in Patients with Inflammatory Bowel Disease. Surg. Endosc..

[9] Bedrikovetski S., Dudi-Venkata N., Kroon H.M., Liu J., Andrews J.M., Lewis M., Lawrence M., Sammour T. (2019). Systematic Review of Rectal Stump Management during and after Emergency Total Colectomy for Acute Severe Ulcerative Colitis. ANZ J. Surg..

[10] Böhm G., O’Dwyer S.T. (2007). The Fate of the Rectal Stump after Subtotal Colectomy for Ulcerative Colitis. Int. J. Color. Dis..

[11] Gu J., Stocchi L., Remzi F., Kiran R.P. (2013). Intraperitoneal or Subcutaneous. Dis. Colon Rectum.

[12] Brady R.R.W., Collie M.H.S., Ho G.T., Bartolo D.C.C., Wilson R.G., Dunlop M.G. (2008). Outcomes of the Rectal Remnant Following Colectomy for Ulcerative Colitis. Color. Dis..

[13] Trickett J.P., Tilney H.S., Gudgeon A.M., Mellor S.G., Edwards D.P. (2005). Management of the Rectal Stump after Emergency Sub-Total Colectomy: Which Surgical Option Is Associated with the Lowest Morbidity?. Color. Dis..

[14] Pal S., Sahni P., Pande G.K., Acharya S.K., Chatttopadhyay T.K. (2005). Outcome Following Emergency Surgery for Refractory Severe Ulcerative Colitis in a Tertiary Care Centre in India. BMC Gastroenterol..

[15] Lawday S., Leaning M., Flannery O., Summers S., Antoniou G.A., Goodhand J., Bethune R., Antoniou S.A. (2020). Rectal Stump Management in Inflammatory Bowel Disease: A Cohort Study, Systematic Review and Proportional Analysis of Perioperative Complications. Tech. Coloproctol..

[16] Buchs N.C., Bloemendaal A.L.A., Wood C.P.J., Travis S., Mortensen N.J., Guy R.J., George B.D. (2017). Subtotal Colectomy for Ulcerative Colitis: Lessons Learned from a Tertiary Centre. Color. Dis..

[17] Lissel M., Omidy S., Myrelid P., Block M., Angenete E. (2020). The Handling of the Rectal Stump Does Not Affect Severe Morbidity After Subtotal Colectomy For Ulcerative Colitis: A Retrospective Cohort Study. Scand. J. Surg..

[18] Schineis C., Lehmann K.S., Lauscher J.C., Beyer K., Hartmann L., Margonis G.A., Michel J., Degro C.E., Loch F.N., Speichinger F. (2020). Colectomy with Ileostomy for Severe Ulcerative Colitis-Postoperative Complications and Risk Factors. Int. J. Color. Dis..

[19] Renaud M., Ayav A., Caron B., Peyrin-Biroulet L., Germain A. (2022). Is Hartmann’s Pouch an Option in the Management of Acute Severe Ulcerative Colitis?. J. Clin. Med..

[20] Ritter A.S., Dumm N., Deisenhofer J.M., Franz C., Al-Saeedi M., Büchler M.W., Schneider M. (2024). Risk Factors for Rectal Stump Leakage after Discontinuity Resection: Stump Length Matters Most. Dis. Colon Rectum.

[21] Huber H., Greenstein A.J., Kayal M., Plietz M.C. (2021). Rectal Stump Leaks in Patients Undergoing Subtotal Colectomy for Ulcerative Colitis: Inflammatory Bowel Disease Center Experience. J. Am. Coll. Surg..

[22] Kumar R., Fernandez L.M., Krizzuk D., Dasilva G., Wexner S.D. (2018). Management of Colorectal Stump after Colectomy: What Matters? A Comparison between IBD and Non-IBD Patients. Dis. Colon Rectum.

[23] Ozcimen E., Hameed I., Tursun N., Yilmaz S., Valente M.A., Steele S., Gorgun E. (2022). Single-Incision Laparoscopic Clockwise Continuous Total Abdominal Colectomy with End Ileostomy for Ulcerative Colitis. J. Am. Coll. Surg..

[24] Frasson M., Pinkney T., Gisbert J.P., Rodriguez-Lago I., Blackwell S., Gecse K.B., Buskens C.J., Knowles C., Zmora O., Brookes M. (2023). P447 Early Predictors of the Need for Surgery in Patients with Acute Severe Ulcerative Colitis: Results of the Prospective, Observational, International ESCP MASC Study. J. Crohns Colitis.

[25] Zaman S., Mohamedahmed A.Y.Y., Abdelrahman W., Abdalla H.E., Wuheb A.A., Issa M.T., Faiz N., Yassin N.A. (2024). Minimally Invasive Surgery for Inflammatory Bowel Disease: A Systematic Review and Meta-Analysis of Robotic Versus Laparoscopic Surgical Techniques. J. Crohns Colitis.

[26] Spinelli A., Bonovas S., Burisch J., Kucharzik T., Adamina M., Annese V., Bachmann O., Bettenworth D., Chaparro M., Czuber-Dochan W. (2022). ECCO Guidelines on Therapeutics in Ulcerative Colitis: Surgical Treatment. J. Crohns Colitis.

[27] Chaudhri S., Rooney P.S. (2008). Surgical Management of Inflammatory Bowel Disease. Surgery.

[28] Karch L.A., Bauer J.J., Gorfine S.R., Gelernt I.M. (1995). Subtotal Colectomy with Hartmann’s Pouch for Inflammatory Bowel Disease. Dis. Colon Rectum.

[29] Fleshner P.R., Michelassi F., Rubin M., Hanauer S.B., Plevy S.E., Targan S.R. (1995). Morbidity of Subtotal Colectomy in Patients with Severe Ulcerative Colitis Unresponsive to Cyclosporin. Dis. Colon Rectum.

[30] Cohen J.L., Strong S.A., Hyman N.H., Buie D.W., Dunn G.D., Ko C.Y., Fleshner P.R., Stahl T.J., Kim D.G., Bastawrous A.L. (2005). Practice Parameters for the Surgical Treatment of Ulcerative Colitis. Dis. Colon Rectum.

[31] Brown S.R., Fearnhead N.S., Faiz O.D., Abercrombie J.F., Acheson A.G., Arnott R.G., Clark S.K., Clifford S., Davies R.J., Davies M.M. (2018). The Association of Coloproctology of Great Britain and Ireland Consensus Guidelines in Surgery for Inflammatory Bowel Disease. Color. Dis..

[32] Magro F., Gionchetti P., Eliakim R., Ardizzone S., Armuzzi A., Barreiro-de Acosta M., Burisch J., Gecse K.B., Hart A.L., Hindryckx P. (2017). Third European Evidence-Based Consensus on Diagnosis and Management of Ulcerative Colitis. Part 1: Definitions, Diagnosis, Extra-Intestinal Manifestations, Pregnancy, Cancer Surveillance, Surgery, and Ileo-Anal Pouch Disorders. J. Crohns Colitis.

[33] Holubar S.D., Lightner A.L., Poylin V., Vogel J.D., Gaertner W., Davis B., Davis K.G., Mahadevan U., Shah S.A., Kane S.V. (2021). The American Society of Colon and Rectal Surgeons Clinical Practice Guidelines for the Surgical Management of Ulcerative Colitis. Dis. Colon Rectum.

[34] Moran G.W., Gordon M., Sinopoulou V., Radford S.J., Darie A.-M., Vuyyuru S.K., Alrubaiy L., Arebi N., Blackwell J., Butler T.D. (2025). British Society of Gastroenterology Guidelines on Inflammatory Bowel Disease in Adults: 2025. Gut.

[35] Lavryk O.A., Holubar S.D. (2023). Rectal Stump Management in IBD. Dis. Colon Rectum.

[36] Ward S.T., Mytton J., Henderson L., Amin V., Tanner J.R., Evison F., Radley S. (2018). Anti-TNF Therapy Is Not Associated with an Increased Risk of Post-colectomy Complications, a Population-based Study. Color. Dis..

[37] Kim J.Y., Zaghiyan K., Lightner A., Fleshner P. (2020). Risk of Postoperative Complications among Ulcerative Colitis Patients Treated Preoperatively with Vedolizumab: A Matched Case-Control Study. BMC Surg..

[38] Alves Martins B.A., Shamsiddinova A., Alquaimi M.M., Worley G., Tozer P., Sahnan K., Perry-Woodford Z., Hart A., Arebi N., Matharoo M. (2024). Creation of an Institutional Preoperative Checklist to Support Clinical Risk Assessment in Patients with Ulcerative Colitis (UC) Considering Ileoanal Pouch Surgery. Frontline Gastroenterol..

[39] Wasmann K.A., van der Does de Willebois E.M., Koens L., Duijvestein M., Bemelman W.A., Buskens C.J. (2021). The Impact of Rectal Stump Inflammation After Subtotal Colectomy on Pouch Outcomes in Ulcerative Colitis Patients. J. Crohns Colitis.

[40] Mahadevan U., Seow C.H., Barnes E.L., Chaparro M., Flanagan E., Friedman S., Julsgaard M., Kane S., Ng S., Torres J. (2025). Global Consensus Statement on the Management of Pregnancy in Inflammatory Bowel Disease. Clin. Gastroenterol. Hepatol..

[41] Nielsen O.H., Gubatan J.M., Kolho K.-L., Streett S.E., Maxwell C. (2024). Updates on the Management of Inflammatory Bowel Disease from Periconception to Pregnancy and Lactation. Lancet.

[42] Luceri C., Femia A., Pietro F.M., Di Martino C., Zolfanelli F., Dolara P., Tonelli F. (2016). Effect of Butyrate Enemas on Gene Expression Profiles and Endoscopic/Histopathological Scores of Diverted Colorectal Mucosa: A Randomized Trial. Dig. Liver Dis..

[43] Korelitz B.I., Cheskin L.J., Sohn N., Sommers S.C. (1984). Proctitis after Fecal Diversion in Crohn’s Disease and Its Elimination with Reanastomosis: Implications for Surgical Management. Report of Four Cases. Gastroenterology.

[44] Frisbie J.H., Ahmed N., Hirano I., Klein M.A., Soybel D.I. (2000). Diversion Colitis in Patients with Myelopathy: Clinical, Endoscopic, and Histopathological Findings. J. Spinal Cord Med..

[45] Ten Hove J.R., Bogaerts J.M.K., Bak M.T.J., Laclé M.M., Meij V., Derikx L.A.A.P., Hoentjen F., Mahmmod N., van Tuyl S.A., Oldenburg B. (2019). Malignant and Nonmalignant Complications of the Rectal Stump in Patients with Inflammatory Bowel Disease. Inflamm. Bowel Dis..

[46] Shen B. (2016). The Evaluation of Postoperative Patients with Ulcerative Colitis. Gastrointest. Endosc. Clin. N. Am..

[47] Komorowski R.A. (1990). Histologic Spectrum of Diversion Colitis. Am. J. Surg. Pathol..

[48] Warren B.F., Shepherd N.A., Bartolo D.C., Bradfield J.W. (1993). Pathology of the Defunctioned Rectum in Ulcerative Colitis. Gut.

[49] Rice A.J., Abbott C.R., Mapstone N.M. (1999). Granulomatous Vasculitis in Diversion Procto-Colitis. Histopathology.

[50] Chetty R., Hafezi S., Montgomery E. (2009). An Incidental Enterocolic Lymphocytic Phlebitis Pattern Is Seen Commonly in the Rectal Stump of Patients with Diversion Colitis Superimposed on Inflammatory Bowel Disease. J. Clin. Pathol..

[51] Derikx L.A.A.P., Nissen L.H.C., Smits L.J.T., Shen B., Hoentjen F. (2016). Risk of Neoplasia After Colectomy in Patients With Inflammatory Bowel Disease: A Systematic Review and Meta-Analysis. Clin. Gastroenterol. Hepatol..

[52] Georganta I., McIntosh S., Boldovjakova D., Parnaby C.N., Watson A.J.M., Ramsay G. (2023). The Incidence of Malignancy in the Residual Rectum of IBD Patients after Colectomy: A Systematic Review and Meta-Analysis. Tech. Coloproctol..

[53] Abdalla M., Landerholm K., Andersson P., Andersson R.E., Myrelid P. (2017). Risk of Rectal Cancer After Colectomy for Patients With Ulcerative Colitis: A National Cohort Study. Clin. Gastroenterol. Hepatol..

[54] Lutgens M.W.M.D., van Oijen M.G.H., Vleggaar F.P., Siersema P.D., Broekman M.M.T.J., Oldenburg B. (2012). Dutch Initiative on Crohn and Colitis Risk Factors for Rectal Stump Cancer in Inflammatory Bowel Disease. Dis. Colon Rectum.

[55] Shen B., Kochhar G.S., Navaneethan U., Cross R.K., Farraye F.A., Iacucci M., Schwartz D.A., Gonzalez-Lama Y., Schairer J., Kiran R.P. (2021). Endoscopic Evaluation of Surgically Altered Bowel in Inflammatory Bowel Disease: A Consensus Guideline from the Global Interventional Inflammatory Bowel Disease Group. Lancet Gastroenterol. Hepatol..

[56] Gordon H., Biancone L., Fiorino G., Katsanos K.H., Kopylov U., Al Sulais E., Axelrad J.E., Balendran K., Burisch J., de Ridder L. (2023). ECCO Guidelines on Inflammatory Bowel Disease and Malignancies. J. Crohns Colitis.

[57] Braithwaite E., Carbonell J., Kane J.S., Gracie D., Selinger C.P. (2021). Patients’ Perception of Colonoscopy and Acceptance of Colonoscopy Based IBD Related Colorectal Cancer Surveillance. Expert Rev. Gastroenterol. Hepatol..

[58] Liao C.-H., Chen P.-J., Shih Y.-L., Chang W.-K., Hsieh T.-Y., Huang T.-Y. (2025). Factors Affecting Perception and Acceptance of Colonoscopy in Patients with Inflammatory Bowel Disease. Prev. Med. Rep..

[59] Napolitano D., Lo Cascio A., Bozzetti M., Povoli A., Grubissa S., Molino L., Marino M., Berretti D., Puca P., Lavigna D.I.R. (2025). Patient Satisfaction with IBD Undergoing Colonoscopy: A Multicenter Cross-Sectional Study. J. Clin. Med..

[60] Ries N.M., Jansen J. (2021). Physicians’ Views and Experiences of Defensive Medicine: An International Review of Empirical Research. Health Policy.

[61] Levesque B.G., Sandborn W.J., Ruel J., Feagan B.G., Sands B.E., Colombel J.-F. (2015). Converging Goals of Treatment of Inflammatory Bowel Disease from Clinical Trials and Practice. Gastroenterology.

[62] Kamp K.J., Hawes S.E., Tse C.S., Singh S., Dang N., Oberai R., Weaver S.A., Melmed G.Y., Siegel C.A., van Deen W.K. (2023). Concordance and Discordance Between Patient-Reported Remission, Patient-Reported Outcomes, and Physician Global Assessment. Inflamm. Bowel Dis..

[63] Calvet X., Ferrario M.G., Marfil V., Armenteros S., Barreiro-de Acosta M. (2025). Patient-Reported Outcome Measures Poorly Correlate with Objective Inflammatory Bowel Disease Activity Measures: A Systematic Review. J. Crohns Colitis.

[64] Sninsky J.A., Barnes E.L., Zhang X., Long M.D. (2022). Urgency and Its Association with Quality of Life and Clinical Outcomes in Patients with Ulcerative Colitis. Am. J. Gastroenterol..

[65] Nigam G.B., Limdi J.K., Bate S., Hamdy S., Vasant D.H. (2024). Fecal Urgency in Ulcerative Colitis: Impact on Quality of Life and Psychological Well-Being in Active and Inactive Disease States. Clin. Gastroenterol. Hepatol..

